# Supplementary material for: Feasibility of implementing the World Health Organization case management guideline for possible serious bacterial infection among young infants in Ntcheu district, Malawi
Source: PLoS One. 2020 Apr 14;15(4):e0229248. doi: 10.1371/journal.pone.0229248 (PMC7156088; doi:10.1371/journal.pone.0229248)
Supplement: S1 Table — (DOCX) [file pone.0229248.s002.docx]

**S1 Table. Timeline of TYIIN study milestones**

| **Study milestone/event** | **Timeline** |
| --- | --- |
| **Policy dialogue and protocol development** |  |
| - Agreement with MoH IMCI unit to conduct implementation research on WHO guideline for case management of PSBI in young infants | April 2015 |
| - Approval from Ntcheu district health office to conduct implementation research on WHO guideline | July 2015 |
| - Research protocol approved by NHSRC | September 2015 |
| **Study preparation and stakeholder engagement** |  |
| - Workshop on WHO guideline for WHO demonstration sites, Ibadan, Nigeria* | November 2015 |
| - District Health Management Team (DHMT) briefing on TYIIN study | March 2016 |
| - Stakeholder and community engagement – District Executive Committee (DEC) meeting in Ntcheu district with district and community leaders | April 2016 |
| - Formation of district-based study support team | May 2016 |
| - Training of master trainers in WHO guideline and TYIIN protocol | June 2016 |
| - Review and finalization of study SOPs, training materials and data collection tools | July 2016 |
| - Initial training of 55 health facility clinicians and nurses from 12 study facilities | July-August 2016 |
| - Training of 146 HSAs and refresher training of facility staff | September 2016 |
| - Orientation of 321 secret mothers | October 2016 |
| - Readiness assessment of 12 study facilities | October 2016 |
| - Final procurement and preparations | November 2016 |
| **Study implementation** |  |
| - Pilot implementation | December 2016-February 2017 |
| - Full implementation | February 22-September 13, 2017 (dates of treatment initiation) |
| **Analysis and dissemination** |  |
| Preliminary data cleaning and analysis | September – November 2017 |
| Dissemination of findings by MoH:   - TYIIN study implementation review meeting, Balaka, Malawi - RAcE multi-country dissemination workshop, Abuja, Nigeria - PSBI implementation research writing workshop, WHO, Geneva - PSBI management implementation research dissemination workshop, Addis Ababa, Ethiopia | September 2017  October 2017  November 2017  January 2018 |
| **Malawi was not an official WHO PSBI demonstration site and was funded under a different mechanism, but efforts were made to align the design and SOPs to be consistent with the other study sites.* | |
