# Supplementary material for: Feasibility of implementing the World Health Organization case management guideline for possible serious bacterial infection among young infants in Ntcheu district, Malawi
Source: PLoS One. 2020 Apr 14;15(4):e0229248. doi: 10.1371/journal.pone.0229248 (PMC7156088; doi:10.1371/journal.pone.0229248)
Supplement: S2 Table — (DOCX) [file pone.0229248.s003.docx]

**S2 Table. Summary of roles and responsibilities for TYIIN study**

| **Role** | **Responsibilities** |
| --- | --- |
| **Technical and implementation support** | |
| **WHO** | - Guide design and development of study standard operating procedures (SOPs) and tools - Conduct monitoring and quality assurance of the study - Help respond to implementation issues and problem solve |
| **Save the Children** | - Design and oversee study implementation, including recruitment and supervision of TYIIN study staff - Technical support for data management and analysis |
| **National IMCI unit** | - Lead adaptation of WHO guideline for Malawi - Provide technical oversight and monitoring of study implementation - Dissemination of study findings |
| **District-based study support team*** | - Participate in adaptation of SOPs and training materials and study tools - Deliver training on WHO guideline and study SOPs to health facility staff and HSAs jointly with TYIIN study staff - Regular supervision and monitoring of implementation at study facilities alongside TYIIN study staff |
| **Full-time TYIIN Study Staff** | |
| **Study Manager (1)** | - Overall responsible for research implementation, in collaboration with investigators - Train study staff and facility staff on study procedures, together with MoH and district-based team - Coordinate and supervise tracking and follow-up of cases, including adverse events - Verify informed consent and study outcomes for sample of cases - Supervise study staff and provide regular updates to study investigators |
| **Clinical Monitor (2)** | - Regular monitoring through visits to health facilities to assess adherence to assessment criteria and availability of study supplies - Conduct home visits to verify data, follow-up on infants who fail to complete required follow-up visits, and collect information on patient satisfaction, and follow-up of severe illness cases and cases who accept referral |
| **Data Manager (1)** | - Ensure data integrity for the study, reviewing data collection and tracking tools for completeness, reviewing and responding to database queries - Managing the process of data entry and cleaning for completed case report forms, and preparing relevant reports for study |
| **Comprised of 13 members including maternal and child health program coordinators, IMCI master trainings, clinical staff from the district hospital and the hospital research coordinator* | |
